# Entanglement-enhanced quantum metrology with neutral atom arrays

**DOI:** 10.1093/nsr/nwaf149

**Published:** 2025-04-17

**Authors:** Heng Shen, Jing Zhang

**Affiliations:** State Key Laboratory of Quantum Optics Technologies and Devices, and Collaborative Innovation Center of Extreme Optics, Shanxi University, China; State Key Laboratory of Quantum Optics Technologies and Devices, and Collaborative Innovation Center of Extreme Optics, Shanxi University, China

## Abstract

This perspective reviews recent progresses in the generation of metrologically useful atomic entangled states with neutral atom array and illustrate a future blueprint for the optimal metrology.

Reconfigurable atom arrays with Rydberg interaction enable both global many-body interactions and gate-based quantum circuits, offering opportunities for large-scale quantum information processing. In this paper, we review the recent progress in the generation of metrologically useful atomic entangled states with neutral atom arrays. We discuss the challenges for quantum metrology, particularly scaling to large atom arrays, entangling gate fidelity and measurement of the dynamic range, as well as the potential future pathways to overcome these obstacles. We also illustrate a future blueprint in terms of a synergistic combination of two distinct avenues to obtain the optimal metrology.

The aim of high-precision metrology is to reduce uncertainties and obtain the best possible conclusion from measurement data. Here, a fundamental limit in precision is the quantum projection noise that results from the inherently probabilistic quantum measurement of *N* identical uncorrelated particles and the associated measurement uncertainty scales as $1/\sqrt N $, known as the standard quantum limit (SQL). This limit, however, can be overcome by exploiting quantum entangled states. In particular, entanglement in atomic systems such as spin-squeezed states and Greenberger–Horne–Zeilinger (GHZ) states offers the opportunity to surpass the SQL and even approach the quantum-theory-allowed precision-bound Heisenberg limit with *1/N* scaling, leading to improvement in the sensitivities of atomic spectroscopy, interferometry and optical atomic clocks. Hence, the building of scalable atomic systems with many-particle entanglement is a major endeavor in quantum metrology.

The neutral atom array is a promising candidate for scalable and programmable quantum many-body platforms. It allows the control and detection of the quantum state of individual quantum objects that interact with each other through Rydberg blockade [1]. Recent years have witnessed great progress in quantum simulation and computation in this platform, especially in logical quantum processors. On the other hand, quantum metrology with neutral atom arrays, as an encouraging approach, is still in its infancy. In contrast with other physical platforms, reconfigurable atom arrays in combination with a dipole–dipole interaction enable both global many-body interactions and gate-based quantum circuits with high controllability. The dipole–dipole interaction leads to van der Waals interaction or spin-exchange interaction, i.e. dipole XY interaction, depending on two atoms prepared in the same or two different Rydberg states. Consequently, two distinct avenues were developed very recently to generate metrologically useful atomic entangled states.

Over the past year, Kaufman, Endres [2,3] and their collaborators have demonstrated a gate-based circuit approach to preparing GHZ states with a few qubits for entanglement-enhanced tweezer optical strontium clocks. In fact, N-qubit GHZ states could accumulate phase N times faster than the associated coherent atomic states, and in theory could saturate the Heisenberg limit. Both teams took full advantage of both programmable atom arrays and optical atomic clocks. The former applied the multi-qubit gate through global Rydberg excitation in the ensembles to prepare the GHZ states, like a one-axis twisting Hamiltonian. The largest ensemble size to which the multi-qubit gate can be operated is determined by the number of atoms that can be placed in a single Rydberg blockade radius. In [2], the authors demonstrated fractional frequency uncertainties at or below the 10^–18^ level while the atom-laser-frequency comparison below the SQL was observed. Specifically, to overcome the limitations of dynamic range with single-size GHZ states and approach the Heisenberg-limit scaling of clock stability at the optimal dark time, they leveraged the strategy of multi-ensemble phase estimation by performing the phase estimation over an extended interval with a cascade of variously sized GHZ states. In contrast, Endres *et al.* used the high-fidelity two-qubit entangling gates up to 99.5% and reconfigurable architectures via atom movement for a Rydberg-gate design to prepare the GHZ states [3]. Importantly, they implemented non-destructive measurements of the clock qubit via ancilla qubit measurement and resetting, as well as shelving optical qubits into superpositions of motional states that were long-lived and dark to imaging light.

Regarding the global interaction-based route, a breakthrough in 2024 [4] provided a generalized theory of scalable spin squeezing of locally interacting systems. The authors conclusively answered the question of whether scalable squeezing can be achieved in the absence of all-to-all interactions, while Rey *et al.* discussed the generation of spin squeezing with short-range interactions [5]. Significantly, Yao *et al.* gave a necessary condition for scalable squeezing that systems must manifest finite-temperature continuous symmetry breaking, which inherently exhibit long-range connected correlations [6]. This intriguing correspondence between finite-temperature order and spin squeezing can be directly applied to Rydberg tweezer arrays with a 2D dipolar XY model. They have reported scalable squeezing with ≤100 rubidium atoms in a Rydberg atom tweezer array within this framework. Also, Kaufman *et al.* used the Rydberg dressing through a van der Waals potential to prepare spin-squeezed states with ^88^Sr atoms [7].

In contrast to other platforms, scalability to larger arrays is one of the unique capabilities of Rydberg atom arrays. Conventionally, defect-free arrays of atoms are assembled by initially stochastically loading up to a single atom into each of a set of tweezers and subsequently rearranging the atoms within the traps. Consequently, increasing the number of optical tweezers is the typical way to boost the scaling. Recently, Endres *et al.* reported the realization of an array of optical tweezers that trapped >6100 neutral atoms in ∼12 000 sites by using two high-power lasers at far-off-resonant wavelengths [8] while they demonstrated a record coherence time for hyperfine qubits with a specially designed room-temperature vacuum chamber. However, they found that high power induced thermal heating of the objective, which worsens optical aberrations, and thus it is not a sustainable method. Moreover, the number of atoms in the final arrays is no greater than the number initially loaded. In this regard, iterative assembly of atom arrays with cavity-enhanced optical lattices [9] may serve as a sustainable solution by repeatedly filling the reservoir with fresh atoms.

Entangling gate fidelity is a critical issue for the circuit-based approach, especially in cascade phase estimation and multi-Rydberg-gate circuits. In 2023, Lukin *et al.* reported two-qubit entangling gates with 99.5% fidelity on ≤60 ^87^Rb atoms in parallel with long-lived m_F_ = 0 hyperfine qubits [10], in which parameterized time-optimal gates were implemented and Rydberg states and other experimental parameters such as intermediate-state detuning were carefully chosen. As for the alkali earth atoms, Thompson *et al.* realized two-qubit gates with fidelities of 0.980 by using the nuclear spin of a long-lived metastable state in ^171^Yb [11] and this record was updated recently by Atom Computing [12] to be 99.72% (99.5) with (without) post-selection with ground-state nuclear spin. In addition, as aforementioned, [3] demonstrated two-qubit entangling gates with 99.62% fidelity averaged over symmetric input states for optical clock qubits of ^88^Sr. Better cooling, dynamical decoupling in atom movement and even error correction can be employed in the near term to improve the circuit performance.

Spin-squeezed states with a large number of atoms entail joint sensitivity to errors and reduced dynamic range, meaning that the large reduction in entanglement induces metrological gain. In addition, challenges to generate large-scale and high-quality spin-squeezed states arise in terms of initial state preparation and measurement error [6,7], decoherence from laser noise [6], inhomogeneity of optical tweezer intensity and Rydberg excitation, and black-body radiation for the Rydberg dressing. A variational algorithm [13] is thus a good strategy for enabling optimal usage of entanglement and optimal sensor designs with finite dynamic ranges even on noisy and non-universal present-day quantum hardware. In this protocol, low-depth, parametrized quantum circuits are used by applying the cost function associated with the Bayesian mean squared error of the estimated phase for a given prior distribution. Also, robust dynamic Hamiltonian engineering [14] is an interesting avenue for preparing the optimal entanglement resource for specific sensing tasks.

On the other hand, due to the characteristics of Rydberg states, black-body radiation induces undesired transitions to nearby strongly interacting Rydberg states, causing Rydberg gates to deteriorate. One solution is to operate in a cryogenic environment. In addition, similarly to a trapped ion system in a cryogenic environment, improvement of the atom trapping lifetime has been observed [15] due to the significant reduction in background gas collision.

In practice, the measurement can be usually divided into three distinct sections: the preparation of a probe, its interaction with the system to be measured and the probe readout. From a broader perspective, we expect a synergistic combination of a global many-body interaction and a gate-based circuit, and apply to these measurement sections complementary functionalities to optimize the metrological gain, as shown in Fig. 1.

**Figure 1.**
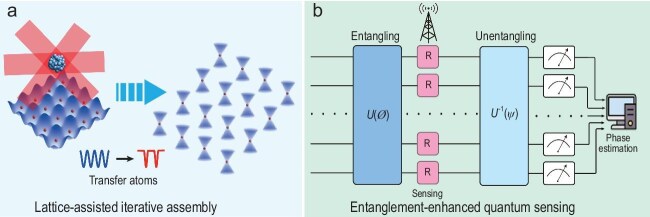
Entanglement-enhanced quantum sensing with neutral atom arrays. (a) Optical lattice-assisted iterative assembly is employed to fill the reservoir with fresh atoms repeatedly and construct the defect-free atom array for implementing the gate-based circuit. (b) In the variational quantum algorithm circuit, global many-body interaction via robust dynamical Hamiltonian engineering is applied to operate the entangling and disentangling gates with variational parameters $\phi $ and $\psi $. Joint phase estimation is used to obtain the optimal estimation.
